# On the Determination of Kappa Distribution Functions from Space Plasma Observations

**DOI:** 10.3390/e22020212

**Published:** 2020-02-13

**Authors:** Georgios Nicolaou, George Livadiotis, Robert T. Wicks

**Affiliations:** 1 Department of Space and Climate Physics, Mullard Space Science Laboratory, University College London, Dorking, Surrey RH5 6NT, UK; r.wicks@ucl.ac.uk; 2 Southwest Research Institute, San Antonio, TX 78238, USA; george.livadiotis@swri.org

**Keywords:** space plasma, kappa distribution, methods, statistical analysis

## Abstract

The velocities of space plasma particles, often follow kappa distribution functions. The kappa index, which labels and governs these distributions, is an important parameter in understanding the plasma dynamics. Space science missions often carry plasma instruments on board which observe the plasma particles and construct their velocity distribution functions. A proper analysis of the velocity distribution functions derives the plasma bulk parameters, such as the plasma density, speed, temperature, and kappa index. Commonly, the plasma bulk density, velocity, and temperature are determined from the velocity moments of the observed distribution function. Interestingly, recent studies demonstrated the calculation of the kappa index from the speed (kinetic energy) moments of the distribution function. Such a novel calculation could be very useful in future analyses and applications. This study examines the accuracy of the specific method using synthetic plasma proton observations by a typical electrostatic analyzer. We analyze the modeled observations in order to derive the plasma bulk parameters, which we compare with the parameters we used to model the observations in the first place. Through this comparison, we quantify the systematic and statistical errors in the derived moments, and we discuss their possible sources.

## 1. Introduction

The velocity distribution function (VDF) of space plasma particles contains all the information we need in order to understand the kinetic and thermodynamic properties of the plasma. Several studies have shown that the VDFs of space plasma particles are kappa distribution functions ([1,2,3,4,5,6,7] and references therein), which consist of a lower energy “core” and a higher energy “tail”. Over the last few decades, several studies have used kappa distribution functions to describe plasma particles in several space regions such as, the solar wind (e.g., [8,9,10,11,12,13,14,15]), planetary magnetospheres (e.g., [16,17,18,19,20,21]), in the vicinity of a comet [22], and the inner and outer heliosheath (e.g., [23,24,25,26,27,28,29]). In the theoretical framework, the kappa distribution function minimizes the Tsallis entropic form under the constraints of the canonical ensemble [30,31,32], which is shown to be the only physically meaningful entropic form consistent with thermodynamics [33].

The three dimensional (3D) isotropic kappa VDF (e.g., [3,6] and references therein) is

(1) $$f(\vec{u})=n\cdot \frac{\Gamma \left(\kappa +1\right)}{\Gamma \left(\kappa -1/2\right)}\cdot {\left[\frac{m}{2\pi k_{B}\left(\kappa -3/2\right)T}\right]}^{3/2}\cdot {\left[1+\frac{m{\left(\vec{u}-{\vec{u}}_{0}\right)}^{2}}{2k_{B}\left(\kappa -3/2\right)T}\right]}^{-\kappa -1},$$

where $\vec{u}$ is the particle velocity vector, *n*, ${\vec{u}}_{0}$, and *T* are the plasma density, bulk velocity vector, and temperature respectively, Γ is the gamma function, *m* the mass of the particle species, and *k*_B_ is the Boltzmann constant. Finally, *κ* is the kappa index that labels and governs the VDF. In order to describe accurately space plasmas, we need high-quality measurements which allow the accurate determination of the VDF. State-of-the-art instruments, such as top-hat electrostatic analyzers, are capable of measuring plasma particle fluxes in velocity space, constructing the 3D VDFs of the plasma particles. Due to technological limitations associated with the instrument’s resolution, range, and efficiency, the 3D VDFs are not always perfectly resolved. Inaccuracies in the measurements can lead to inaccurate description of the plasma. Furthermore, the total error of the derived plasma parameters also depends on the method we use to analyze the observations [34,35,36,37].

We highlight the importance of the accurate determination of *κ,* which describes the thermodynamic distance from the classic thermal equilibrium (e.g., [38]) and is related with the correlation between the plasma particles (e.g., [3,39]). Interestingly, recent studies have shown that the kappa index is related with the polytropic index of space plasmas [21,33,40,41], which must be determined for the valid characterization and understanding of physical mechanisms, such as transitions through shocks [42,43,44,45], plasma turbulent compressions (e.g., [36,46]), particle collisions [47], and many more. Importantly, previous studies demonstrated that inaccurate estimations of the kappa index can lead to significant misestimations of other plasma bulk parameters [2,3,34].

**Figure 1:**
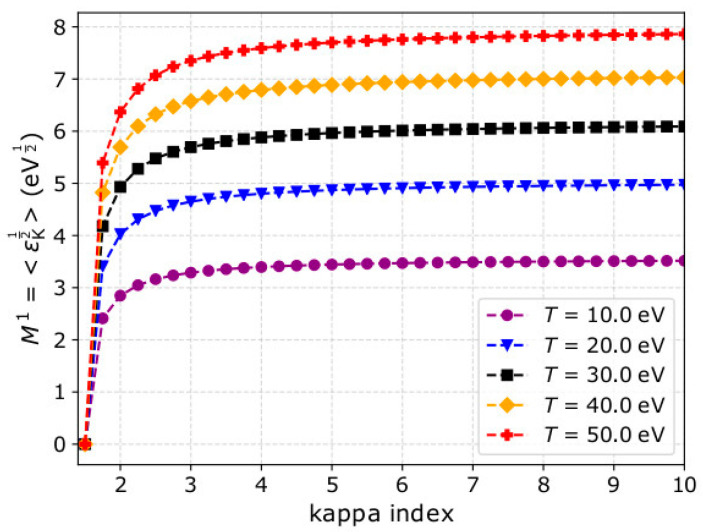
The first order kinetic energy moment $M^{1}$ as a function of the kappa index *κ*, for five different plasma temperatures *T*.

Typical analysis of the VDF calculates the velocity moments of the VDF via numerical integration. From the different orders of the velocity moments, we determine the plasma density, bulk velocity, and temperature. However, there is no velocity moment that is a function of the kappa index. Instead, the speed (or kinetic energy) moments of the VDF are functions of the temperature *T* and the kappa index *κ*. Livadiotis [6] and [48] have derived the kinetic energy moments of the kappa distribution function

(2) $$M^{a}\equiv \langle \varepsilon_{Κ}^{\frac{1}{2}\alpha }\rangle ={\left(k_{B}T\right)}^{\frac{1}{2}\alpha }\kappa_{0}{}^{\frac{1}{2}\alpha }\cdot \frac{\Gamma \left(\frac{d_{Κ}}{2}+\frac{\alpha }{2}\right)}{\Gamma \left(\frac{d_{Κ}}{2}\right)}\cdot \frac{\Gamma \left(\kappa_{0}+1-\frac{\alpha }{2}\right)}{\Gamma \left(\kappa_{0}+1\right)},$$

where $\varepsilon_{Κ}=\frac{1}{2}m{\left(\vec{u}-{\vec{u}}_{0}\right)}^{2}$ is the kinetic energy of the plasma particles in the reference frame of the bulk flow, *α* is the order of the moment, and *d*_K_ denotes the kinetic degrees of freedom. In Equation (2) we use the notation of the invariant kappa index $\kappa_{0}\equiv \kappa -\frac{d_{k}}{2}$ (for more details see [48,49]). As explicitly shown by [6], only the 0th (*α* = 0) and the 2nd (*α* = 2) order *ε*_Κ_ moments do not depend on *κ*, while any other order is a combination of *κ* and *T*. In this study we examine 3D VDFs, therefore *d*_K_ = 3, and $\kappa =\kappa_{0}+\frac{3}{2}$. In this consideration, *κ* ranges between 3/2 and ∞. According to Equation (2) and as discussed in [6], only moments of order α ≤ 2 converge for all possible *κ* values. For instance, the first order moment (*α* = 1) for a 3D VDF is

(3) $$M^{1}=\langle \varepsilon_{Κ}^{\frac{1}{2}}\rangle ={\left(k_{B}T\right)}^{\frac{1}{2}}\cdot \frac{2}{\sqrt{\pi }}\cdot {\left(\kappa -\frac{3}{2}\right)}^{\frac{1}{2}}\cdot \frac{\Gamma \left(\kappa -1\right)}{\Gamma \left(\kappa -\frac{1}{2}\right)},$$

and Figure 1 shows *M*^1^ as a function of *κ*, for five different temperatures. For all the temperatures we show, there is a sharp increase of $M^{1}$ as a function of *κ* within the range 1.5 < *κ* < 4, and a plateau for *κ* > 4. The numerical calculation of Equation (3) leads to the determination of *κ*. Such a novel calculation could be useful for future analyses and/or could be applied on-board in future operations for fast estimations. However, we firstly need to validate this method considering plasma measurements with realistic uncertainties, obtained by an instrument with realistic detection efficiency, field of view, and resolution.

The purpose of this paper is to demonstrate and quantify the derivation of the kappa index for distributions constructed from plasma measurements. In order to do that, we model observations of typical solar wind plasma protons with their velocities following the isotropic kappa distribution function, considering a realistic response of an electrostatic analyzer. We then analyze the observations by constructing the 3D VDFs from the observations and calculate the statistical moments that allow the calculation of the plasma parameters. The difference between the derived and the input plasma parameters quantifies the accuracy of the specific method for the specific instrument design and plasma conditions. In the next section, we show how we construct our synthetic solar wind observations and how we construct the VDF and analyze it to obtain the statistical moments. In Section 3, we show the results for a synthetic solar wind plasma, and we quantify the accuracy of the derived parameters. In Section 4, we discuss in detail our results, and in Section 5 we summarize our conclusions.

## 2. Methods 

### 2.1. Synthetic Data Set

We use the forward modeling method (e.g., [20,34,35,36,37,50,51,52,53,54,55,56]) to simulate solar wind proton observations by a typical top-hat electrostatic analyzer with an electrostatic aperture deflector system and a position-sensitive Multi-Channel-Plate (MCP) detector. Our model instrument measures protons within the energy range from 200 ev to 20 keV, in 96 electrostatic steps of the electrostatic analyzer, each with resolution Δ*E*/*E* ~ 5%. The instrument resolves the elevation direction of the particles *Θ* within the range from −22.5° to +22.5°, in 9 electrostatic scans of the aperture deflector. Each elevation angle is resolved with resolution Δ*Θ* = 5°. The MCP resolves the azimuth direction of the particles *Φ* within the range from −45° to +45°, in 16 azimuth sectors with resolution Δ*Φ* = 6°. 

We model solar wind protons with velocities following the isotropic kappa distribution function. The instrument scans through the energies and directions of the particles in discrete *Ε*, *Θ*, *Φ* bins and registers the amount of particles that hit the MCP detector within the acquisition time Δ*τ* and produce detectable signal. For this study, we use Δ*τ* = 1 millisecond. The expected amount of detected particles is approximately

(4) $$C(E,\Theta ,\Phi )=\frac{2}{m^{2}}GE^{2}f(E,\Theta ,\Phi )\Delta \tau ,$$

where *m* is the mass of the proton and $G=A_{0}\frac{\Delta Ε}{Ε}\Delta \Theta \Delta \Phi$ is the instrument’s geometric factor with *A*_0_ the effective aperture, which is a function of the geometric aperture and the detection efficiency (for more see [35,36,37]). For our study, we use $A_{0}=4.4\times 10^{-6}$ m^2^, and combined with the energy resolution and the solid angle covered by the instrument’s angular resolution results to $G=2\times 10^{-9}$ m^2^∙eV/eV∙sr. Although Equation (4) gives the expected average number of detected particles for each *E*, *Θ*, *Φ* pixel, in reality, the registered counts *C*_out_ follow the Poisson distribution function of average *C*(*E*,*Θ*,*Φ*), with measurement probability

(5) $$P(C_{\mathrm{out}})=e^{-C}\frac{C^{C_{out}}}{C_{\mathrm{out}}!}.$$

**Figure 2:**
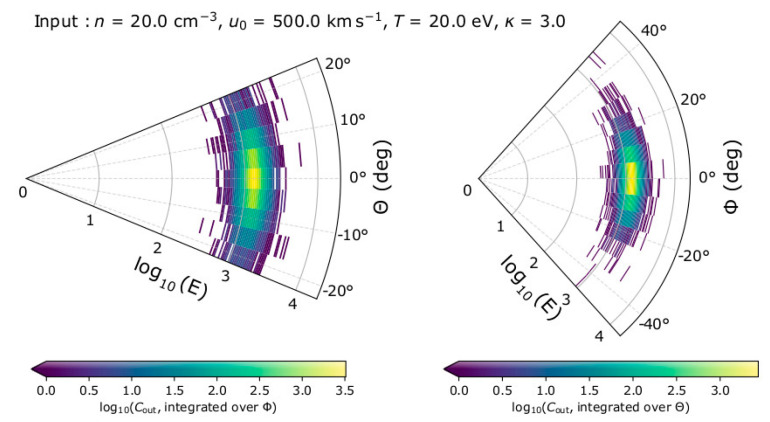
Measurement sample for plasma with *n* = 20 cm^−3^, $u_{0}$ = 500 kms^−1^ towards *Θ* = 0° and *Φ* = 0°, *T* = 20 eV, and *κ* = 3, recorded by the top-hat electrostatic analyzer design we consider in this study. The left panel shows the registered number of counts *C*_out_ as a function of log_10_(*E*) and *Θ* integrated over *Φ*, while the right panel shows *C*_out_ as a function of log_10_(*E*) and *Φ*, integrated over *Θ*.

In the left panel of Figure 2, we show one example of registered counts as a function of energy and elevation angle, integrated over the azimuth angles. In the right panel of Figure 2, we show the registered counts as a function of energy and azimuth, integrated over the elevation angles. For the specific example, we model plasma with *n* = 20 cm^−3^, $u_{0}$ = 500 kms^−1^ with direction towards *Θ* = 0° and *Φ* = 0°, *T* = 20 eV and *κ* = 3.

### 2.2. Statistical Moments

In plasma applications, we usually consider that *C*_out_ (*E,Θ,Φ*) ~ *C*(*Ε*,*Θ,Φ*) and the kinetic energy distribution function is constructed from the observations, using the inverse of Equation (4):

(6) $$f_{\mathrm{out}}(E,\Theta ,\Phi )=\frac{m^{2}C_{\mathrm{out}}\left(Ε,\Theta ,\Phi \right)}{2G{Ε}^{2}\Delta \tau },$$

from which we can obtain the VDF $f_{\mathrm{out}}(\vec{u})$ for $u=\sqrt{\frac{2E}{m}}$. Commonly, the plasma bulk parameters are determined from the velocity moments of *f*_out_, i.e., the plasma density

(7) $$n_{\mathrm{out}}=\int f_{\mathrm{out}}(\vec{u})d^{3}u,$$

the plasma bulk velocity vector

(8) $${\vec{u}}_{0,\mathrm{out}}=\frac{1}{n_{\mathrm{out}}}\left[\left(\int (\vec{u}\cdot \hat{x})f_{\mathrm{out}}(\vec{u})d^{3}u\right)\hat{x}+\left(\int (\vec{u}\cdot \hat{y})f_{\mathrm{out}}(\vec{u})d^{3}u\right)\hat{y}+\left(\int (\vec{u}\cdot \hat{z})f_{\mathrm{out}}(\vec{u})d^{3}u\right)\hat{z}\right],$$

and the elements of the temperature tensor

(9) $$T_{\mathrm{out}}^{ij}=\frac{1}{n_{\mathrm{out}}k_{B}}\int mw_{ij}^{2}f_{\mathrm{out}}(\vec{u})d^{3}u,$$

where, with *i* and *j* running through the x, y, and z components. Finally, the $w_{ij}=\left(u_{i}-u_{0j,\mathrm{out}}\right)$ scalar temperature is determined as

(10) $$T_{\mathrm{out}}=\frac{1}{3}\left(T_{\mathrm{out}}^{\mathrm{xx}}+T_{\mathrm{out}}^{\mathrm{yy}}+T_{\mathrm{out}}^{\mathrm{zz}}\right).$$

The *α* order kinetic energy moment is

(11) $$M_{\mathrm{out}}^{\alpha }=\frac{1}{n_{\mathrm{out}}}{\int \left[\frac{1}{2}m{\left(\vec{u}-{\vec{u}}_{0,\mathrm{out}}\right)}^{2}\right]}^{\frac{\alpha }{2}}f_{\mathrm{out}}\left(\vec{u}\right)d^{3}u.$$

and according to Equation (2), the kappa index *κ*_out_ is determined by solving

(12) $$M_{\mathrm{out}}^{\alpha }={\left(k_{B}T_{\mathrm{out}}\right)}^{\frac{1}{2}\alpha }{\left(\kappa_{\mathrm{out}}-\frac{3}{2}\right)}^{\frac{1}{2}\alpha }\cdot \frac{\Gamma \left(\frac{3}{2}+\frac{\alpha }{2}\right)}{\Gamma \left(\frac{3}{2}\right)}\cdot \frac{\Gamma \left(\kappa_{\mathrm{out}}-\frac{1}{2}-\frac{\alpha }{2}\right)}{\Gamma \left(\kappa_{\mathrm{out}}-\frac{1}{2}\right)}.$$

Having completed the set of the statistical moments in Equations (7)–(11), we determine the complete set of the plasma bulk parameters. Here we focus on the derivation of the *κ*_out_ by numerically solving Equation (12). The accurate derivation of *κ*_out_, depends on the accuracy of *T*_out_ and $M_{\mathrm{out}}^{\alpha }$, which we examine through this paper.

## 3. Results

**Figure 3:**
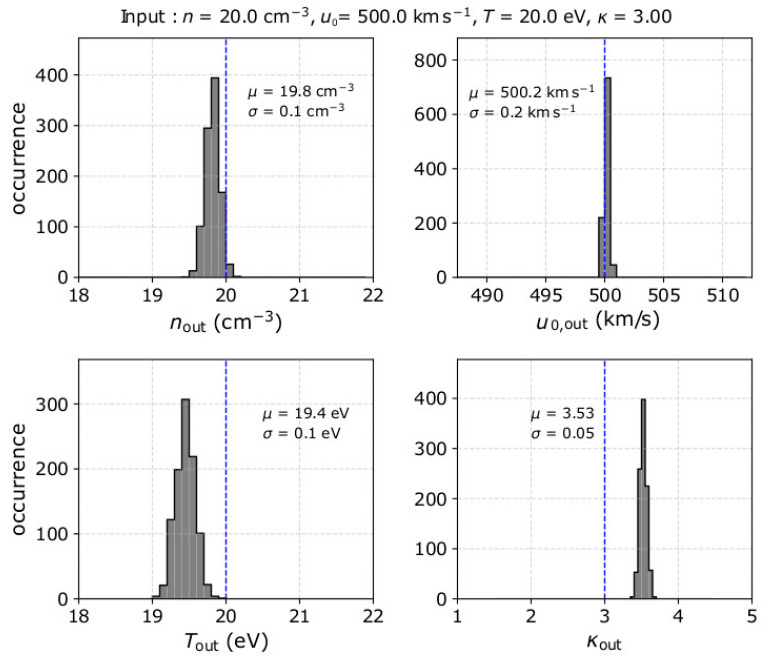
Histograms of the derived (**top left**) *n*_out_, (**top right**) *u*_0,out_, (**bottom left**) *T*_out_, and (**bottom right**) *κ*_out_, by the analysis of 1000 measurement samples considering plasma with *n* = 20 cm^−3^, $u_{0}$ = 500 kms^−1^ towards *Θ* = 0° and *Φ* = 0°, *T* = 20 eV, and *κ* = 3. In each panel, we show the mean value *μ* and the standard deviation *σ* of the derived moments, while the vertical blue dashed line indicates the corresponding input value.

We examine the accuracy of the derived moments for plasma with *n* = 20 cm^−3^, $u_{0}$ = 500 kms^−1^ towards *Θ* = *Φ* = 0°, *T* = 20 eV, and *κ* = 3, which are typical solar wind proton parameters within the heliocentric distance range from 0.3 to 1au (e.g., [57,58]). We model 1000 observation samples for the specific input parameters. We analyze each sample as explained in Section 2.2 in order to determine *n*_out_, *u*_0,out_, *T*_out_, and *κ*_out_. In Figure 3, we show the histograms of the derived plasma parameters for the 1000 modeled observation samples. In the example shown in Figure 3, we calculate *κ*_out_ from the first order energy moment $M_{\mathrm{out}}^{1}$ (α = 1). On average, the analysis of the specific plasma underestimates the plasma density and temperature and overestimates the kappa index. More specifically, the average *n*_out_ is ~19.8 cm^−3^, which is by ~1% smaller than the actual *n*. The average *T*_out_ is 19.4 eV, which is by ~3% smaller than the actual *T*. The average *κ*_out_ is about 3.5, while the actual *κ* = 3. Finally, we find that the average plasma speed does not deviate from the actual value.

Our results in Figure 3, indicate that in addition to the systematic error, the plasma parameters are derived within a certain standard deviation. We specifically calculate σ*_n_*_,out_ ~ 0.1 cm^−3^, σ*_u_*_0_*,*_out_ ~ 0.2 kms^−1^, σ*_T_*_,out_ ~ 0.1 eV, and σ*_κ_*_,out_ ~ 0.05. The total error of the derived plasma parameters (statistical and systematic) depends on the plasma input and the accuracy with which the instrument measures the particle flux (e.g., [34,35,36,37]). In Section 4, we discuss further the sources of errors.

**Figure 4:**
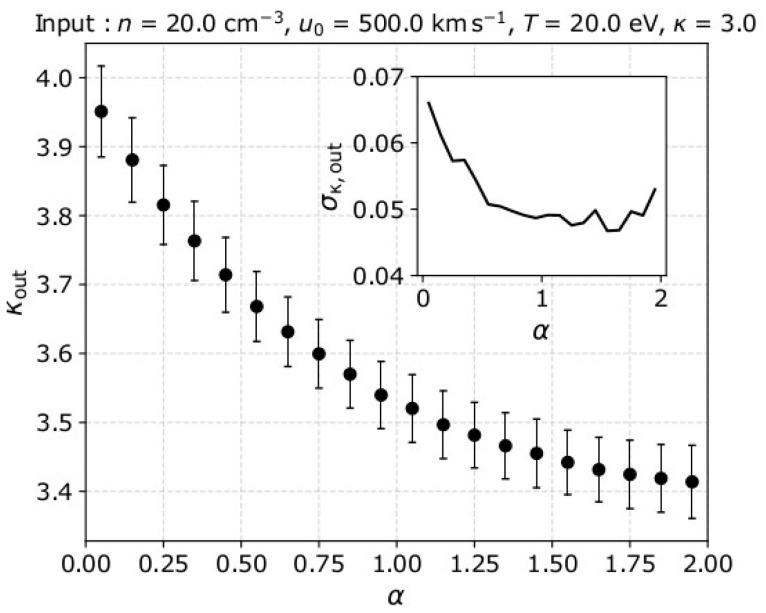
The mean kappa index *κ*_out_ and its standard deviation σ_κ,out_ as functions of the energy moment order we use to analyze the data-set.

We would also like to examine the accuracy of *κ*_out_ as calculated from the kinetic energy moments of different orders *α*. In Figure 4, we show the average *κ*_out_ and its standard deviation, as functions of *α* for the same input plasma parameters as in the example in Figure 3. We investigate the results for *α* values within 0 and 2, which are the boundaries of the converging energy moment orders (see also [6] and references therein). For each α value, we analyze 1000 samples following the Poisson distribution in Equation (5). The derived kappa index *κ*_out_ ~ 3.95 for α→0 and *κ*_out_ ~ 3.4 for α→2. The standard deviation of the mean *κ*_out_ values is σ_κ,out_ ~ 0.07 for α→0 and reduces to σ_κ,out_ ~ 0.05 for α→2. In the next section we discuss in detail our results. 

## 4. Discussion

We demonstrate the analysis of plasma measurements that estimates the kappa index from the statistical moments of the velocity distribution function of the plasma particles. The analysis of the synthetic solar wind proton data sets in our study shows that the specific method systematically overestimates the kappa index.

**Figure 5:**
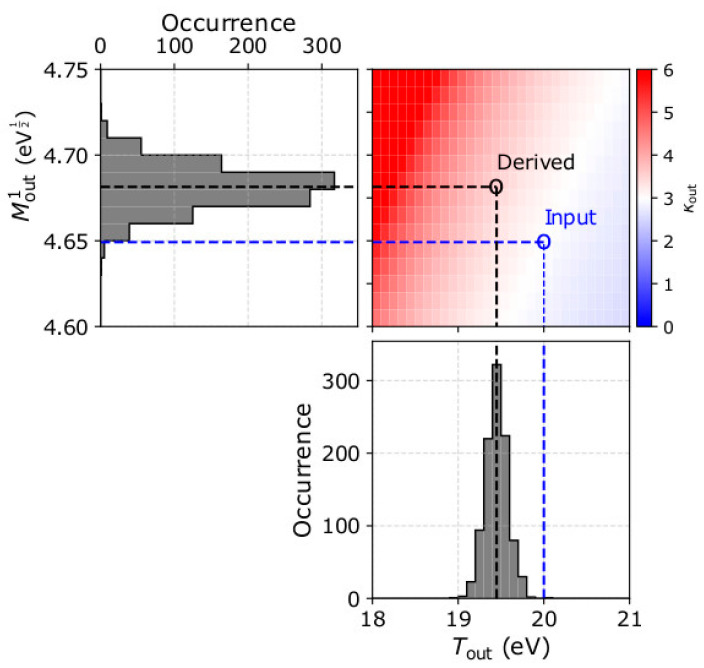
(**Top left**) The occurrence of $M_{\mathrm{out}}^{1}$ and (**lower right**) *T*_out_, as derived from the analysis of 1000 samples of plasma with *n* = 20 cm^−3^, $u_{0}$ = 500 kms^−1^ towards *Θ* = 0° and *Φ* = 0°, *T* = 20 eV, and *κ* = 3. (**Top right**) Solutions of *κ*_out_ as a function of *T*_out_ and $M_{\mathrm{out}}^{1}$ according to Equation (12). On each panel, the blue lines indicate the input parameters and the black lines the derived parameters in our example.

In fact, the kappa index is calculated by numerically solving Equation (12). Thus, an accurate calculation of *κ*_out_ is based on the accuracy of *T*_out_ and $M_{\mathrm{out}}^{\alpha }$. Any systematic error of *T*_out_ and/or $M_{\mathrm{out}}^{\alpha }$ results in a systematic error of *κ*_out_. In Figure 5, we examine the values of *κ*_out_ as a function of *T*_out_ and the first order energy moment $M_{\mathrm{out}}^{1}$. The top left panel shows the histogram of $M_{\mathrm{out}}^{1}$ and the lower right panel the histogram of *T*_out_ as derived from the analysis of 1000 samples considering plasma protons with *n* = 20 cm^−3^, $u_{0}$ = 500 kms^−1^ towards *Θ* = 0° and *Φ* = 0°, *T* = 20 eV, and *κ* = 3. In the top right panel, we show the solution matrix for *κ*_out_ as a function of *T*_out_ and $M_{\mathrm{out}}^{1}$, as calculated from Equation (12). On the same matrix, we indicate the input and the average derived parameters in our analysis. The derived *κ*_out_ in our example is overestimated due to the misestimation of *T*_out_ by ~3% and the overestimation of $M_{\mathrm{out}}^{1}$ by just 0.5%. 

The misestimation of the statistical moments is due to the instrument’s limited efficiency, energy and angular range, energy and angular resolution, and poor statistics related to the sampling of the distribution function in discrete steps (e.g., [34,35,36,37]). For instance, the instrument’s limited efficiency prevents the detection of low particle fluxes which are allowing the construction of the high energy tails of a distribution function. Additionally, there are cases when the distribution function drifts beyond the instrument’s energy and angular range. In these cases, Equations (7) and (9) underestimate the plasma density and temperature respectively, as *f*_out_ is under-sampled. Moreover, plasma instruments resolve the distribution function in finite Δ*Ε*, Δ*Θ*, and Δ*Φ* intervals. As a result, the shape of the actual distribution within individual Δ*Ε*, Δ*Θ*, and Δ*Φ* pixels and its contribution to the statistical moments cannot be quantified. Similarly, the distribution is sampled in discrete energy and angular steps, and the statistical moments are numerically calculated according to the specific limited sampling (binning).

Importantly, we expect the accuracy to depend on all the plasma bulk parameters, as they affect the shape of the VDF e.g., [35,36,37]. For instance, plasmas with higher temperatures have broader VDFs with bigger portion of their tails drifting beyond the instrument’s angular range, causing an underestimation of *n*_out_ and *T*_out_. On the other hand, colder plasmas have narrower VDFs, which are harder to sample with a limited angular resolution. In another example, plasmas with higher densities will increase the number of recorded counts, therefore will reduce the statistical (Poisson) error. The detailed characterization of the accuracy as a function of the plasma parameters is beyond the scope if this study but will be the subject of a future project.

We note that several missions apply moments calculation algorithms on-board spacecraft to enable fast calculations and anticipate the limited telemetry. The specific method we demonstrate here provides novel estimations of the kappa index, which completes the set of the plasma bulk parameters. Moreover, we note that such a method is useful for on-ground calculations in any application beyond plasma VDFs, where kappa distributions play a significant role (e.g., [59,60]). However, users of this method should be aware of the potential errors exposed in this study and use similar approach for their quantification.

**Figure 6:**
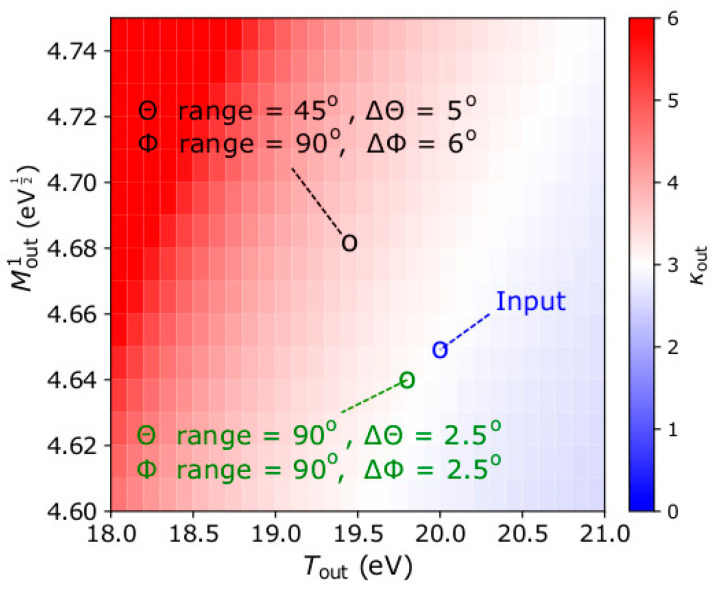
Solutions of *κ*_out_ as a function of *T*_out_ and $M_{\mathrm{out}}^{1}$ according to Equation (12). The black circle indicates the average parameters as derived from the analysis of 1000 observation samples by our standard instrument model with field of view −22.5° < *Θ* < +22.5°, −45° < *Φ* < +45°, and angular resolution Δ*Θ* = 5° and Δ*Φ* = 6° respectively. The green circle indicates the average parameters as derived from the analysis of 1000 observation samples by an instrument with field of view −45° < *Θ* < +45°, −45° < *Φ* < +45°, and angular resolution Δ*Θ* = Δ*Φ* = 2.5°. The input plasma parameters are the same as in Section 3 and are indicated by the blue circle.

Finally, we demonstrate how the accuracy depends on the instrument’s field of view and resolution. We do that by analyzing the same plasma as in Section 3 considering two different instrument designs; one as described in Section 2, and a second one with double the *Θ* range (−45° < *Θ* < +45°) and with better angular resolution (Δ*Θ* = Δ*Φ* = 2.5°). In Figure 6, we show the results of the analysis in the same format as in the top right panel of Figure 5. As expected, the analysis of the observations by the second instrument design calculates more accurately the plasma moments. The improved angular resolution minimizes numerical errors associated with the limited sampling of the VDF’s shape, while the increased field of view captures a bigger portion of the distribution function which contributes to the statistical moments.

## 5. Conclusions

We demonstrate the derivation of plasma bulk parameters by calculating the statistical velocity and kinetic energy moments of a modeled kappa distribution as constructed from the observations by a typical electrostatic analyzer. We apply the mathematical tools demonstrated by [6,48] to simulated observations and we quantify the accuracy of the plasma parameters when derived from the specific method. Our analysis shows that:

- The velocity moments of the observed distribution underestimate the plasma density and temperature, but they provide an accurate estimation of the plasma bulk speed.
- The calculation of the kinetic energy moments of order between 0 and 2 leads to the estimation of the kappa index value. The accuracy of the derived index value is slightly improved as the order of the used energy moment increases. Nevertheless, due to instrument limitations, the analysis systematically overestimates the kappa index of the plasma.
- The misestimations of the plasma parameters are due to the instrument’s limited efficiency, energy and angular range, resolution, and limited sampling of the actual plasma distribution. Our analysis quantifies the error of the derived parameters for a specific instrument design and plasma conditions. Similarly, future applications could quantify the expected errors by adjusting the instrument and plasma parameters. Moreover, our results could drive future instrument designs in order to achieve the desired accuracy in specific applications.
